# Does LLLT improve radiographic healing for dogs with cranial cruciate ligament rupture undergoing TPLO surgery?

**DOI:** 10.18849/ve.v10i4.726

**Published:** 2025-11-05

**Authors:** Lucy Moore+

**Keywords:** CANINE, CRANIAL CRUCIATE LIGAMENT RUPTURE, LOW-LEVEL LASER THERAPY, ORTHOPAEDIC, RADIOGRAPHIC BONE HEALING, TIBIAL PLATEAU LEVELLING OSTEOTOMY

## Abstract

**PICO question:**

In canine patients undergoing tibial plateau levelling osteotomy (TPLO) surgery for unilateral cranial cruciate ligament rupture (CCLR), is LLLT (low-level laser therapy (less than 200 mw)) treatment effective at reducing time to radiographic bone healing compared to no LLLT treatment?

**Clinical bottom line:**

**Category of research:**

Treatment.

**Number and type of study designs reviewed:**

Three studies (study 1: randomised, blinded, prospective clinical study, study 2: randomised, double blinded, placebo-controlled, parallel-group clinical trial and study 3: randomised controlled trial).

**Strength of evidence:**

Weak.

**Outcomes reported:**

In all three studies the authors compared the use of LLLT to a control and concluded that LLLT treatment did not make a significant difference in improving radiographic bone healing. Therefore, the evidence which suggests LLLT improves radiographic bone healing in dogs recovering from TPLO surgery is weak.

**Conclusion:**

There is a lack of conclusive evidence surrounding the use of LLLT treatment in dogs who underwent TPLO surgery. Based on current data it is difficult to say whether LLLT is beneficial and this demonstrates the requirement for further study to truthfully determine whether the laser device is effective for radiographic bone healing. One of three studies suggests that LLLT is beneficial to canine patients undergoing TPLO surgery for CCLR, radiographic bone healing in dogs was not the focus of that review.

## How to apply this evidence in practice

The application of evidence into practice should take into account multiple factors, not limited to: individual clinical expertise, patient’s circumstances and owners’ values, country, location or clinic where you work, the individual case in front of you, the availability of therapies and resources.

Knowledge Summaries are a resource to help reinforce or inform decision-making. They do not override the responsibility or judgement of the practitioner to do what is best for the animal in their care.

## Clinical scenario

A canine patient suffering from cranial cruciate ligament rupture (CCLR) is brought by their owner to your clinic, which is known for performing routine tibial plateau levelling osteotomy (TPLO) surgeries. As the operating veterinary surgeon you wish to consider if low-level laser therapy (LLLT) would be beneficial in improving radiographic bone healing following the surgery and if the procedure should be included in your postoperative care protocol.

## The evidence

After the exclusion criteria was applied, three studies (three randomised controlled trials) (Kennedy et al., 2018; Renwick et al., 2018; Rogatko et al., 2017) were appraised and are included in this Knowledge Summary. All three studies investigated the impact the use of LLLT (low-level laser therapy) had on radiographic bone healing in canine patients following a TPLO surgery. All three studies are randomised controlled trials. The overall strength of evidence is weak. All three studies show no benefit of LLLT on radiographic bone healing in dogs post TPLO surgery.

## Summary of the evidence

**Kennedy et al. (2018):** Effects of low-level laser therapy on bone healing and signs of pain in dogs following tibial plateau levelling osteotomy **Aim: **To assess the result of low-level laser therapy (LLLT) on inflammation, signs of pain, function, bone healing and osteoarthritis in dogs following tibial plateau leveling osteotomy (TPLO) who suffered from spontaneous cranial cruciate ligament rupture (CCLR).

Population:	Client owned dogs with unilateral cranial cruciate ligament rupture (CCLR). Dogs included had a spontaneous unilateral CCLR and a stable contralateral stifle joint and were ≥ one year old and weighed ≥ 15kg. Dogs who were evaluated and treated at Washington State University Veterinary Teaching Hospital were considered for enrolment.
Sample size:	12 dogs (6 spayed females, 6 neutered males).
Intervention details:	Dogs were randomly split into 2 groups using a random number generator. Low-level laser therapy treatment (LLLT) group (n = 6) received LLLT. Red light treatment (control) group (n = 6) received red light treatment. Both groups received treatment for 5 minutes immediately before and after surgery and again at 6, 12, 24, 36, 48, 60, 72, 84, and 96 hours after surgery while hospitalised. Once discharged they received treatment for 3 minutes every other day for 4 weeks. While hospitalised the LLLT group was treated with a dual-probe class 2 laser with four 5-mW diodes with a wavelength of 635 nm and a radiant exposure of 2.23 J/cm^2^. After discharge the LLLT group was treated with a customised class 2 laser with one 5-mW diode with a wavelength of 635 nm and a radiant exposure of 1.5 J/cm^2^. The control group was treated with the same laser units as the LLT group with the exception of the 5-mW diodes, which were replaced with red LED lightbulbs. Laser and control treatments were almost identical as both emitted a visual red light.
Study design:	Randomised controlled trial.
Outcome Studied:	Establish the effects of LLLT on markers of synovial inflammation and signs of pain, function, bone healing and osteoarthritis in dogs with spontaneous CCLR following tibial plateau levelling osteotomy (TPLO) surgery.
Main Findings (relevant to PICO question):	The main finding of the study is that the laser protocol used had no beneficial effects on the LLLT group. None of the radiographic variables assessed differed between the 2 groups at any time except for the extent of soft tissue inflammation at 8 weeks after TPLO. This soft issue inflammation affected a greater number of dogs in the laser group than the control group. Pain scores using the modified Glasgow composite pain scoring system did not differ significantly when assessed by surgical team between LLLT and control groups. Mean accelerometric activity did not differ significantly between the LLLT and control groups at any of the measured time points. The findings of this study also suggest that LLLT had detrimental effects, or the control treatment had beneficial effects that exceeded those of the LLLT.
Limitations:	There was a small sample size. Owners were required to keep a treatment journal and complete a Canine Brief Pain Inventory (CBPI) at weekly intervals after surgery. When owners assessed signs of pain and used the CBPI significant differences between the 2 groups were occasionally observed. Furthermore, the dogs in the control group generally had lower pain scores or improved limb function compared to dogs in the LLLT group. The limitation is that any pain scoring system is subjective and can be attributed to owner bias. The LLLT treatment was carried out by owners at home, this could have led to errors in: the placement of the device (over incision site on the medial aspect of the stifle joint) the duration of treatment (3 minutes) the frequency of treatment (every other day for 4 weeks). LLLT may have caused an inhibitory biological response as the pain scores increased in the immediate postoperative period. The lack of radiographical changes may have been due to the short postoperative observation period or small sample size.

**Renwick et al. (2018):** Influence of class IV laser therapy on the outcomes of tibial plateau leveling osteotomy in dogs **Aim: **To assess the effects of low-level laser therapy (LLLT) on clinical outcomes in dogs with cranial cruciate ligament disease who were treated with tibial plateau leveling osteotomy (TPLO) surgery.

Population:	Client owned dogs with unilateral cranial cruciate ligament rupture (CCLR) treated with tibial plateau leveling osteotomy (TPLO). Dogs included had no coexisting disease that could influence their healing or the outcome measures, no contralateral TPLO within the preceding 4 months, and no history of aggression. Dogs who were referred to the clinic for TPLO surgery within the trial dates.
Sample size:	95 dogs.
Intervention details:	Dogs were randomly split into 2 groups. Laser therapy treatment group (LG) (n = 51) received laser therapy. Placebo group (PG) (n = 44) received the placebo. Both groups received treatment to the stifle area, from about L4 to S1. Both groups received treatment on 3 consecutive days within a four day perioperative period, specifically on days -1 (day of admission – pre-op), 0 (day of operation – immediately post-op), +1 (1 day post-op) and +2 (2 days post-op). When TPLO surgery was performed on the same day as admission, treatments were applied on days 0, +1 and +2. When TPLO surgery was performed the day after admission, treatments were applied on days -1, 0 and +1. A fourth treatment was recommended between days 10 and 14 but not compulsory. Laser treatment emitted red light at 660 nm [100 mW] and infrared light at 800, 905, and 970 nm infrared [up to 15 W continuous and 20 W peak], whereas the placebo treatment only emitted red light at 660 nm [4 mW]. Treatment comprised of 10 phases of different pulse frequency: continuous wave followed by 2 Hz, 10 Hz, 50 Hz, 100 Hz, 200 Hz, 500 Hz, 1000 Hz, 5000 Hz and completed with a continuous wave again. Laser and placebo treatments were almost identical to both owner and/or surgeon as both emitted a visual red light and an audible fan sound.
Study design:	Randomised, double-anonymised, placebo-controlled, parallel-group clinical trial.
Outcome Studied:	Influence a laser protocol has on the clinical outcomes of dogs treated with TPLO through measuring the difference in clinical metrology instruments, osteotomy radiographic healing, time to cessation of NSAID administration, and wound healing according to an owner questionnaire.
Main Findings (relevant to PICO question):	The main finding of the study is that the laser protocol did not influence radiographic bone healing in dogs recovering from TPLO. The laser protocol used in the study showed a greater improvement in ACOI (adjusted Canine Orthopaedic Index) gait. ACOI gait improved by 6 units in the LG compared to an improvement of 4 units in the PG. No statistically significant differences between LG and PG. Only variable significantly different between groups was the presence of a postoperative osteotomy gap, which did not occur in the LG group but occurred in 4 of the PG cases.
Limitations:	Groups were slightly unevenly split. Improvement of gait was measured using an owner questionnaire. The number of treatments was reduced from the recommended 6 to 3 or 4 due to the inconveniency of repeated visits from clients travelling long distances to the clinic. Only 27/95 (28.4%) of cases (LG, n = 14 and PG, n = 13) received a fourth day of treatment. Adjustment of the Canine Orthopaedic Index (COI) questionnaire through removing the most frequently unanswered questions and changing the function, gait and total sections before analysis, may have risked the validity of these previously validated outcome measures. The study author did not specify what the ‘units’ they refer to when stating the findings.

**Rogatko et al. (2017):** Preoperative low-level laser therapy in dogs undergoing tibial plateau levelling osteotomy: A blinded, prospective, randomized clinical trial **Aim: **To assess the influence of pre-operative low-level laser therapy (LLLT) on the healing effects of dogs undergoing tibial plateau levelling osteotomy (TPLO).

Population:	Client owned dogs with unilateral cranial cruciate ligament rupture (CCLR) tibial plateau levelling osteotomy (TPLO) surgery.
Sample size:	27 dogs.
Intervention details:	Dogs were randomly split into 2 groups by coin toss. Low-level laser therapy (LLLT) group (n = 12) received an active treatment. Placebo control treatment group (SHAM) (n = 15) received a placebo control treatment. Both groups received treatment to the proximomedial region of the tibia: 1 dose prior to TPLO surgery. The LLLT group received treatment using a gallium-aluminimum-arsenium laser which was 800–900 nm dual wavelength, 6 W, 3.5 J/cm^2^, 100 cm^2^. The LLLT group received laser therapy at a continuous wavelength at 3 watts for 30 seconds, the 2 hz at 4 watts for 45 seconds, then at 5 hz at 4 watts for 30 seconds, than at 10 hz at 3 watts for 45 seconds, then at 500 hz at 3 watts for 30 seconds. Both groups underwent the same postoperative pain management routine which included an injectable opioid for 12–24 hours, tramadol twice a day for 14 days and carprofen twice a day for 14 days. Both groups also received cryotherapy for 5 minutes every 4 hours for the first 24 hours.
Study design:	Randomised, double-anonymised, prospective clinical study.
Outcome Studied:	Influence of LLLT therapy on radiographic bone healing in dogs with CCLR after TPLO surgery.
Main Findings (relevant to PICO question):	There was no difference in radiographic bone healing found between the groups at eight weeks postoperation.
Limitations:	There was a small sample size. Three dogs in the laser group did not show up for the eight-week postoperative assessment. The radiographic scoring system which was used in this study was not specific to dogs who underwent TPLO surgery as it was modified from a human grading system.

## Appraisal, application and reflection

The goal of this Knowledge Summary was to investigate the effectiveness of low-level laser therapy (LLLT) treatment in improving radiographic bone healing in canine patients who suffered from cranial cruciate ligament rupture (CCLR) where tibial plateau levelling osteotomy (TPLO) surgery was recommended. There are a limited number of studies that investigate the use of LLLT in canines for the improvement of radiographic bone healing following a TPLO surgery which was performed due to CCLR.

Three studies that directly investigated the PICO question were found (Kennedy et al., 2018; Renwick et al., 2018; Rogatko et al., 2017). All three were randomised control trials. The current lack of research in this area weakens the level of evidence for this Knowledge Summary and highlights the need for future research. All three of the studies outlined above are reliable and repeatable. There are very few studies which discuss the use of LLLT in animals and even less dealing with orthopaedic issues in dogs.

The first study outlined above by Rogatko et al. (2017) indicates that LLLT treatment compared to a placebo treatment had no significant difference in radiographic bone healing in dogs following uncomplicated TPLO surgery. However, the study groups were extremely small with 12 dogs in the LLLT group and 15 dogs in the placebo group. Additionally, only eight dogs attended the 8-week postoperative recheck in the LLLT group which made the group even smaller.

The 8 week postoperative radiographs revealed that in the LLLT group 5/8 dogs showed signs of osteotomy healing while in the placebo group 3/12 dogs showed signs of osteotomy healing, which was not a significant difference. The study author concluded that a single preoperative dose of LLLT did not cause a significant difference in signs of radiographic bone healing 8 weeks postoperative in comparison to no laser treatment at all (Rogatko et al., 2017). In addition to this Rogatko et al. (2017) carried out a lameness assessment and response to manipulation, and force plate analysis. These were performed preoperation and then 24 hours, 2 weeks, and 8 weeks postoperation. Peak Vertical Force (PVF) and Vertical Impulse (VI) (pressure exerted by each limb) were not significantly different between groups 24 hours and 2 weeks postoperation, however PVF was significantly different 8 weeks postoperation, VI was not significantly different between groups. There was no significant difference in lameness scores at all during the study (Rogatko et al., 2017).

The second study outlined above by Renwick et al. (2018) indicates that LLLT compared to no laser treatment (red light) had no significant difference in bone healing in dogs following uncomplicated TPLO surgery. However, the study groups were split unevenly and take up for a recommended fourth round of treatment was quite low at 27/95 (28.4%) (Renwick et al., 2018). Outcomes were measured by difference in clinical metrology instruments, osteotomy radiographic healing, time cessation of non-steroidal anti-inflammatory drug (NSAID) administration and wound healing through the use of an owner questionnaire. The only difference identified between groups was a greater improvement in the gait section of the ACOI (adjusted Canine Orthopedic Index) in the laser group compared to the placebo group. In the laser group the ACOI gait improved the placebo group at 8 weeks postoperation. The study author did not specify what the ‘units’ they are referring to when stating these findings. This is not a hugely significant difference and the PICO question remains unanswered (Renwick et al. 2018).

Additionally, the third study outlined above by Kennedy et al. (2018) also found that LLLT had no significant difference in radiographic bone healing following uncomplicated TPLO surgery. In this study the author compared LLLT to a control which was red light therapy. In addition, physical and orthopaedic exams, force plate analysis, synoviocentesis of the affected joint, lameness and signs of pain were assessed 2 weeks postoperation, 4 week, and 8 weeks. The author found that there was no significant difference between the two groups regarding: limb function, the synovial fluid assessment, signs of pain. However, when limb function was assessed at 2 weeks and 4 weeks postoperation, the function was poorer in the laser group than the control group. Additionally, when force plate analysis was used the study author found that at 2 weeks and 4 weeks postoperative function of the affected limb was better in dogs in the control group compared to the laser group. Furthermore, when signs of pain were assessed by owners the control group received lower pain scores or improved limb function. As a result, the study author concluded that there was a possibility that LLLT had negatively affected healing or perhaps that the control treatment had beneficial effects that surpassed those of the LLLT (Kennedy et al., 2018). However, the sample size used in this study was extremely small with only 12 dogs and the significant difference in signs of pain were mainly found between the LLLT group and the control group when owners were asked to assess their dogs at home. Furthermore, having owners assess this, resulted in the consistency of data being compromised as the pain scoring system in general is subjective.

All three appraised studies are randomised controlled trials; these sit on the second level on the evidence hierarchy pyramid. However, a limitation is that there is always a risk of bias, as Rogatko et al. (2017) and Kennedy et al. (2018) have very small sizes (27 dogs and 12 dogs respectively), which weakens the overall strength of the evidence. Additionally, whilst the studies are randomised controlled trials, they are subject to owner bias due to the subjective nature of pain scoring systems (Renwick et al., 2018) and owner inconsistencies when performing LLLT treatment (Kennedy et al., 2018).

From the available data, LLLT treatment has not been shown to improve radiographic bone healing in dogs who underwent TPLO surgery. Ultimately, this requires further research as there is a possibility that LLLT shows promise in other areas such as reducing the incidence of surgical site infections, which is highlighted by Chavez et al. (2024). There is no significant difference in radiographic bone healing in the studies outlined in this Knowledge Summary. Considering the weak evidence presented in these studies, patient factors such as age, breed, history, and more must be taken into consideration in the decision making of using LLLT on a case by case basis.

## Methodology

**Search Strategy:** 

Databases searched and dates covered:	CAB Abstracts on EBSCO Platform (2001–2024) PubMed on National Institutes of Health (NIH) (2001–2024)
Search strategy:	CAB Abstracts: (dog* OR canine*) (laser therapy OR laser treatment OR low-level laser therapy OR low-level light therapy OR photobiomodulation OR PBMT OR LLLT OR laser medicine OR local laser therapy) ((reduc* OR improv* OR minimis* OR radiographic OR orthopaedic) AND (bone fracture OR bone heal* OR recover*)) 1 AND 2 AND 3 PubMed: (dog* OR canine*) (laser therapy OR laser treatment OR low-level laser therapy OR low-level light therapy OR photobiomodulation OR PBMT OR LLLT OR laser medicine OR local laser therapy) ((reduc* OR improv*OR minimis* OR radiographic OR orthopaedic) AND (bone fracture OR bone heal* OR recover*)) 1 AND 2 AND 3
Dates searches performed:	23 Nov 2024

**Exclusion / Inclusion Criteria:** 

Exclusion:	Not specifically studying dogs to answer PICO question. Systematic reviews, book chapters, congress papers, and case reports. Use of LLLT treatment for other reasons such as wound healing or dental treatments. Not using radiographs to assess bone healing In vitro studies. Not specific to TPLO surgery Not live patients.
Inclusion:	Controlled clinical trials.

**Search Outcome:** 

Database	Number of results	Excluded – not specific to dogs	Excluded – not relevant to answering the PICO question	Excluded – not in English	Total relevant papers
CAB Abstracts	9	0	5	1	3
PubMed	27	2	20	2	3
Total relevant papers when duplicates removed					3

## Acknowledgements

I would like to thank my lecturers, Naoimh Mc Cann and Karen Dunne, for recommending I enter this competition.

## ORCiD

Lucy Moore: https://orcid.org/0009-0000-6744-8940

## Conflict of Interest

The author declares no conflicts of interest.
